# “Same difference”: comprehensive evaluation of four DNA methylation measurement platforms

**DOI:** 10.1186/s13072-018-0190-4

**Published:** 2018-05-25

**Authors:** Thadeous J. Kacmarczyk, Mame P. Fall, Xihui Zhang, Yuan Xin, Yushan Li, Alicia Alonso, Doron Betel

**Affiliations:** 1 Division of Hematology/Oncology, Department of Medicine, Epigenomics Core Facility, Weill Cornell Medicine, New York, NY USA; 2 Institute for Computational Biomedicine, Weill Cornell Medicine, New York, NY USA

**Keywords:** Bisulfite sequencing, DNA methylation, Methylome capture, RRBS, 5mC, CpG

## Abstract

**Background:**

DNA methylation in CpG context is fundamental to the epigenetic regulation of gene expression in higher eukaryotes. Changes in methylation patterns are implicated in many diseases, cellular differentiation, imprinting, and other biological processes. Techniques that enrich for biologically relevant genomic regions with high CpG content are desired, since, depending on the size of an organism’s methylome, the depth of sequencing required to cover all CpGs can be prohibitively expensive. Currently, restriction enzyme-based reduced representation bisulfite sequencing and its modified protocols are widely used to study methylation differences. Recently, Agilent Technologies, Roche NimbleGen, and Illumina have ventured to both reduce sequencing costs and capture CpGs of known biological relevance by marketing in-solution custom-capture hybridization platforms. We aimed to evaluate the similarities and differences of these four methods considering each platform targets approximately 10–13% of the human methylome.

**Results:**

Overall, the regions covered per platform were as expected: targeted capture-based methods covered > 95% of their designed regions, whereas the restriction enzyme-based method covered > 70% of the expected fragments. While the total number of CpG loci shared by all methods was low, ~ 24% of any platform, the methylation levels of CpGs covered by all platforms were concordant. Annotation of CpG loci with genomic features revealed roughly the same proportions of feature annotations across the four platforms. Targeted capture methods comprise similar types and coverage of annotations and, relative to the targeted methods, the restriction enzyme method covers fewer promoters (~ 9%), CpG shores (~ 8%) and unannotated loci (~ 11%).

**Conclusions:**

Although all methods are largely consistent in terms of covered CpG loci, the commercially available capture methods result in covering nearly all CpG sites in their target regions with few off-target loci and covering similar proportions of annotated CpG loci, the restriction-based enrichment results in more off-target and unannotated CpG loci. Quality of DNA is very important for restriction-based enrichment and starting material can be low. Conversely, quality of the starting material is less important for capture methods, and at least twice the amount of starting material is required. Pricing is marginally less for restriction-based enrichment, and the number of samples that can be prepared is not restricted to the number of capture reactions a kit supports. However, the advantage of capture libraries is the ability to custom design areas of interest. The choice of the technique would be decided by the number of samples, the quality and quantity of DNA available and the biological areas of interest since comparable data are obtained from all platforms.

**Electronic supplementary material:**

The online version of this article (10.1186/s13072-018-0190-4) contains supplementary material, which is available to authorized users.

## Background

DNA cytosine methylation in the form of 5-methylcytosine (5mC) in CpG context is an epigenetic marker that is important for regulation of gene expression. Changes in CpG methylation are implicated in many diseases, and proper methylation patterns are required for normal development [1–8]. Large-scale studies such as ENCODE [9] and the Human Epigenomics Roadmap [10] have performed extensive profiling of 5mC in various cell lines and tissues revealing a rich and dynamic landscape of 5mC patterns in the human genome. Given the importance of these markers to cellular development and contribution to disease, a number of approaches have been developed for detecting the methylation status of cytosines [11], with bisulfite sequencing [BS-seq, massively parallel sequencing after chemical deamination of cytosines (C) to uracils (U), followed by polymerase chain reaction (PCR)] being widely used to provide single-base quantitative measurement of cytosine methyl-modifications (5mC and 5-hydroxymethylcytosine, 5hmC). The deamination of cytosines is accomplished by the use of sodium bisulfite, and this pre-treatment preserves both the methyl-modifications in 5mC and the 5hmC [12]. The benchmark in methylome coverage is whole genome bisulfite sequencing (WGBS), which at 30× sequencing coverage detects ~ 94% of all cytosines in the genome with 99.8% of those detected being CpG loci [13]. The early strategy for preparing WGBS libraries, known as pre-bisulfite conversion sequencing, relied on adding methylated adapters to sonicated DNA followed by bisulfite conversion and PCR amplification. Since bisulfite conversion causes DNA fragmentation and degradation [14], this approach needs high amounts of starting material to produce genomic representative libraries. Thus, methods to optimize this protocol have been sought and are currently in use. Post-bisulfite sequencing techniques, where bisulfite sequencing is performed first and libraries are made by subsequent amplification from single stranded DNA, require less starting material and are now commonly used [15–17]. However, these different WGBS library preparation protocols can bias region coverage due to differences in the bisulfite conversion step, choice of amplification primers and choice of polymerases [18]. Since WGBS can be prohibitively expensive, and no method completely covers the methylome, and biologically relevant CpGs have been identified in known genomic features [1, 19], developing focused assays considering these aspects are in demand with the caveat that these approaches will leave gaps in the methylome potentially excluding important CpGs.

The study of biologically relevant CpG sites was accelerated by the use of tiled microarray platforms, providing an alternative low cost approach to WGBS [20]. This technology requires pre-treatment of the DNA, either by immunoprecipitation of methylated DNA or the use of methylation sensitive/insensitive restriction enzymes, to obtain an enriched CpG-fraction for hybridization. The data obtained provide a global methylation value for each region assessed [21]. Quantitative measurements of DNA methylation can be obtained by using the Illumina Methylation BeadChip array, which use bisulfite conversion and base-specific fluorescence to detect the methylation state [22, 23].

In this study, our aim is to investigate characteristics of next generation sequencing (NGS)-based approaches, which provide quantitative nucleotide methylation levels. There are several NGS-based methods for acquiring DNA methylation levels, and we investigated the characteristics of four currently widely used platforms: (1) enrichment by enzymatic digestion (MspI) enhanced reduced representation bisulfite sequencing (ERRBS) [24, 25], (2) capture-based Agilent SureSelect Methyl-Seq (SSMethylSeq) [26], (3) capture-based Roche NimbleGen SeqCap Epi CpGiant (CpGiant) [27], and (4) Illumina TruSeq-Methyl capture EPIC (TruSeqEpic) [28].

ERRBS is a modification of RRBS [29, 30] where the combination of library preparation modifications and downstream data processing produced increased coverage of genomic regions and increased number of CpGs covered [24]. In ERRBS, the DNA is digested with MpsI and enrichment occurs by size selection of the MspI fragments corresponding to 84–334 bp. This fraction is size-selected from an agarose gel in two fragments: one of 84–184 and one of 185–334, each one is bisulfite converted independently. After PCR amplification, these two fractions are normalized and pooled at the same molarity before sequencing, resulting in good representation of the 84–334 bp fraction [25]. ERRBS yields roughly ~ 10% of genomic CpG sites and provides enrichment in CpG islands and CpG shores, promoters, exons, introns and intergenic regions [24]. SSMethylSeq [26] and TruSeqEpic [28] libraries are made from sonicated DNA and are hybridized against the capture platforms. The enriched DNA fraction is bisulfite converted and PCR amplified before sequencing. By design, these two techniques capture one of the two DNA strands during hybridization, primarily to reduce cost. The SSMethylSeq baits for the human genome, designed in 2012, are biotinylated RNA oligos of ~ 100 bp that comprise 84 Mb and cover 3.7 M biologically relevant CpGs (Additional file 1: Table S2). TruSeqEpic was designed in 2016, as a hybridization capture method to complement and expand Illumina’s Infinium Methylation Epic BeadChip Array [31]. TruSeqEpic contains a DNA oligo pool that comprises 107 Mb and covers 3.3 M biologically relevant CpGs [28] (Additional file 1: Table S2); it is mostly designed to capture CpGs from one strand, except on areas of known SNPs where both strands are present in the platform. It is important to note that for additional applications of the downstream data, any method that captures CpGs from both DNA strands can detect SNPs. The CpGiant capture platform, released in 2014, differs from all other methods in that enrichment is done post-bisulfite conversion and the baits were designed to capture the potential methylation state on both strands. This oligo design is made possible through Roche’s ability to manufacture millions of DNA probes in parallel using their proprietary SeqCap Epi enrichment system. The design encompasses 80.5 Mb and covers both strands of 2.8 M CpGs [27] (Additional file 1: Table S2). Table 1 summarizes the differences in library preparation of each method notably: the enrichment method, pre- or post-hybridization bisulfite conversion, and PCR amplification.

**Table 1:** Protocol comparison

DNA requirement	ERRBS	SSMethylSeq		CpGiant		TruSeqEpic	WGBS-PBAT
	75 ng > 40 kb	1 µg	3 µg	0.25 µg	1 µg	0.5 µg	100 ng > 40 kb
DNA processing	MspI digestion to completion followed by fractionation of 84–334 bp	Sonication to 150–200 bp		Sonication to 180–220 bp		Sonication to 180–220 bp	Single stranded and fragmented during bisulfite conversion
Enrichment method	Size fractionation of 84–334 bp sizes	Hybridization to RNA capture probes		Hybridization to oligo probes containing fully, partially and unmethylated cytosines from both strands		Hybridization to oligo probes of stranded design	None
Bisulfite conversion step	Post-adapter ligation	Post-hybridization capture		Pre-hybridization capture		Post-hybridization capture	Pre-adapter tagging
Zymo Research Bisulfite Conversion kit	EZ DNA Methylation (50 °C, 55 cycles)	EZ DNA Methylation-Gold (64 °C, 2.5 h)		EZ-Methylation Lightning (54 °C, 1 h)		EZ-Methylation Lightning (54 °C, 2 h)	EZ DNA Methylation-Gold
Total PCR amplification cycles	18; Post enrichment and bisulfite conversion	14; Post enrichment and bisulfite conversion (8 for amplification and 6 for Indexing)		29; 13 post-bisulfite conversion and 16 post enrichment	27; 11 post-bisulfite conversion and 16 post enrichment	11; Post enrichment and bisulfite conversion	10; Post-bisulfite conversion
DNA Polymerase (uracil tolerant)	FastStart Taq (Roche)	Taq2000 (Agilent Technologies)		HiFi HotSart Uracil + (Kapa Biosystems)		HiFi HotSart Uracil + (Kapa Biosystems)	FailSafe Enzyme (Epicentre)
Predicted number of targeted CpG sites	6.6 M	3.7 M		5.6 M		3.3 M	56 M
Relative price (library preparation + PE100 sequencing @300 M reads)	13.5%	15.5%		15.5%		3.3% (@75 M reads)	100%

Here we present an analysis of the methylation patterns obtained for human lung fibroblast IMR-90 cell line using each of the platform protocols. We chose IMR90 cells because it is a widely studied cell line, commonly used as control DNA by both researchers and companies and it can be obtained from the American Type Culture Collection (ATCC) [13]. The rationale for the libraries made for each platform was as follows. For ERRBS, we prepared two technical duplicates, based on data generated on our previous papers which indicate a level of noise [24, 25]. The commercial platforms require µg amounts of input material, which can be a limiting factor for clinical applications. We tested the impact of the amount of input material on the final results with SSMethylSeq and CpGiant since they are amenable to reducing the input. Thus, the two libraries prepared for each of SSMethylSeq and CpGiant, one at the manufacturer’s suggested concentration (3 and 1 µg, respectively) and one at a reduced concentration (1 and 0.25 µg, respectively). TruSeqEpic libraries were prepared as a multiplex of 4 independently barcoded libraries going into the capture step, as per manufacturer’s recommendations (0.5 µg), and because of platform constrains, we were unable to test lower inputs. Libraries for ERRBS, SSMethylSeq and CpGiant were sequenced to equivalent depth, TruSeqEpic libraries, due to the 4-plex constraint, were sequenced as per manufacturer’s recommendation which resulted in less sequencing depth. To achieve equivalent sequencing depth for TruSeqEpic samples, the data from two duplicate libraries were combined into a single sample. We made one WGBS library using the post-bisulfite adapter tagging kit from EpiCentre (WGBS-PBAT). For economic reasons, the WGBS-PBAT library was sequenced to 10× sequencing depth. (Table 1 and Additional file 1: Table S1). All libraries were compared to the WGBS-PBAT library.

## Methods

### Cell growth and DNA preparation

IMR90 cells (American Type Culture Collection, Manassas, VA cat # CCL-186) were provided by Dan Hasson (Mount Sinai School of Medicine, New York, NY). DNA from 5 × 10^7^ cells was purified using the Gentra Puregene DNA kit according to manufacturer protocol (cat # 158389, Qiagen Valencia, CA). DNA was resuspended in TE, quantified using fluorometric quantification (Qubit 2.0 ThermoFisher Scientific Waltham, MA), and quality was assessed by running on a 1% agarose gel.

### ERRBS (digestion-based enhanced reduced representation bisulfite sequencing)

Two ERRBS libraries (ERRBS_A, ERRBS_B) were prepared as described in Garrett-Bakelman, et al. [25]. Briefly, 75 ng of DNA was digested with the methylation insensitive MspI enzyme (C^CGG). After end-repair, A-tailing, and adapter ligation with Illumina TruSeq adapters, the region corresponding to 84–334 bp was size-selected as two fractions. Each fraction was subjected to overnight bisulfite conversion (55 cycles of 95 °C for 30 s, 50 °C for 15 min) using EZ DNA methylation kit (Cat # D5002, Zymo Research, Irvine CA). Purified bisulfite converted DNA was PCR-amplified using TruSeq primers (Illumina Inc. San Diego, CA) for 18 cycles of denaturing, annealing and extension/elongation steps using Roche FastStart (cat # 03 553 361 001) at 94 °C for 20 s, 65 °C for 30 s, 72 °C for 1 min, followed by 72 °C for 3 min. The resulting libraries were normalized to 2 nM and pooled at the same molar ratio. Libraries were clustered at 6.5 pM on a V3 paired-end read flow cell and sequenced for 100 cycles (PE100) on an Illumina HiSeq 2500.

### Agilent SureSelect Methyl-Seq (SSMethylSeq)

One library (SSMethylSeq_A_opt) made using 3 µg of DNA according to the company’s specifications using manual version B, 2013, (SureSelectXT cat # 5190-4836, Agilent Technologies, Santa Clara CA). DNA (3 µg) was sonicated using a Covaris S220 sonicator (Covaris, Woburn, MA) to obtain products of 150–200 bp. DNA was then end-repaired, A-tailed and ligated with methylated adapters to create a pre-capture DNA library. DNA (500 ng) was then hybridized to the RNA SureSelect Human methyl-seq capture library at 65 °C for 16 h. Hybridized products were purified by capture with Streptavdin beads and then subjected to bisulfite conversion (64 °C for 2.5 h) using the Zymo EZ DNA Gold kit (Cat # D5005, Zymo Research, Irvine CA). The bisulfite-treated libraries were PCR-amplified for 8 cycles with Agilent Taq 2000, after clean-up the library was further PCR-amplified by another 6 cycles to index the library with Illumina Index 6. A second library (SSMethylSeq_B_min) was made using 1 µg of DNA. In this case 250 ng of pre-capture library was used for hybridization, the number of post-hybridization PCR cycles was left the same, and the library was indexed using Illumina Index 12. Each library was clustered at 11 pM on a V3 paired-end read flow cell and sequenced for 100 cycles (PE100) on an Illumina HiSeq 2500.

### Roche NimbleGen SeqCap Epi CpGiant (CpGiant)

One library (CpGiant_A_opt) was made using 1 µg of starting material according to the company’s specifications (SeqCap Epi CpGiant Enrichment kit, cat # 07138881001, Roche, Indianapolis, IN). DNA (1 µg) was sonicated using a Covaris S220 sonicator (Covaris, Woburn, MA) to obtain products of 180–220 bp. DNA was then end-repaired, A-tailed, ligated with methylated indexed-adapters to create a pre-capture DNA library and indexed using Illumina Index 6. The pre-capture library was bisulfite converted at 54 °C for 1 h using the Zymo EZ DNA Lightning kit (Cat # D5030, Zymo Research, Irvine CA). The bisulfite-treated pre-capture library was PCR-amplified for 11 cycles with HiFi HotStart Uracil + polymerase (Cat# KK2802, Kapa Biosystems, Wilmington, MA). 1 µg of the amplified, bisulfite converted library was then hybridized to the probe pool of fully, partially and unmethylated cytosines from both strands of DNA oligos at 47 °C for 72 h. Hybridized products were purified by capture with Capture Beads and PCR amplified for 16 cycles to create the final library. A second library (CpGiant_B_min) was made using 0.25 µg of starting material. DNA was then end-repaired, A-tailed, ligated with methylated indexed-adapters to create a pre-capture DNA library and indexed using Illumina Index 12. The library was then amplified for 13 cycles after post-bisulfite PCR amplification, 800 ng of library were used for hybridization and the post-capture PCR amplification cycles were kept at 16. Each library was clustered at 12 pM on a V3 paired-end read flow cell and sequenced for 100 cycles (PE100) on an Illumina HiSeq 2500.

### TruSeq-Methyl Capture EPIC (TruSeqEpic)

The TruSeqEpic protocol is a 4-plex capture library strategy, where 4 samples are carried through library preparation and are hybridized as one pool (TruSeq-Methyl Capture EPIC, cat # FC-151-1002, Illumina Inc., San Diego, CA). We followed the company’s specifications and made two libraries using 0.5 µg of IMR90 DNA (TruSeqEpic_A, TruSeqEpic_B); the other two libraries were made with DNA from cell line K562 (American Type Culture Collection, Manassas, VA cat # CCL-243, lymphoblast from chronic myelogenous leukemia). DNA was sonicated using a Covaris S220 sonicator (Covaris, Woburn, MA) to obtain products of 180–220 bp. DNA was then end-repaired, A-tailed, ligated with methylated indexed-adapters to create pre-capture DNA libraries. Illumina Indexes 2 and 4 were used for IMR90, Illumina Indexes 5 and 6 for K562. The 4 indexed pre-capture libraries were combined and hybridized to the EPIC oligos twice, at 58 °C for 2 and 14.5 h, respectively. Each time, hybridized products were purified by capture with Streptavidin beads and washed twice to remove nonspecific binding. After the last elution, hybridized products were subjected to bisulfite conversion (54 °C for 2 h) with the reagent provided in the kit. The bisulfite-treated libraries were PCR-amplified for 11 cycles with Kapa HiFi HotStart Uracil + polymerase (Cat# KK2802, Kapa Biosystems, Wilmington, MA). Libraries were clustered at 10 pM on a V3 paired-end read flow cell and sequenced for 100 cycles (PE100) on an Illumina HiSeq 2500.

### Whole genome bisulfite sequencing post-bisulfite adapter tagging (WGBS-PBAT)

100 ng of DNA were bisulfite converted using EZ DNA Methylation-Gold Kit (cat # D5005, Zymo Research Corporation, Irvine, CA) and the single stranded DNA obtained processed for library construction using the EpiGnome Methyl-Seq kit (Cat. # EGMK81312, Illumina Inc., San Diego, CA) as per manufacturer’s protocol. The DNA was made double stranded by the use of 5′ tagged random hexamers and subsequently 3′ tagged with a terminal tagging oligo. The di-tagged DNA was enriched using 10 cycles of PCR, with PCR using a primer containing Illumina Index 12. The library was clustered at 7 pM on a V3 paired-end read flow cell and sequenced for 100 cycles (PE100) on an Illumina HiSeq 2500.

### Description of analysis

The comparison was focused on several features that are characteristic to DNA bisulfite sequencing experiments. These included read alignments, number of CpGs and their methylation levels as well as strand bias. First we evaluated sequencing run performance and alignment consistency. Since the SSMethylSeq and TruSeqEpic platforms are designed to cover predominantly one strand, we devised an even comparison tool across all platforms; thus, we compared strand symmetry of methylation values. Lastly we evaluated several measures to quantify properties of each platform relating to the target regions that are covered, methylation levels of cytosines in CpG context and coverage of genomic regions. Target region analysis included the number of CpGs covered, the fraction of target regions covered, the coverage of target region CpGs, the overlap of CpGs across all platforms, and the concordance of methylation levels of overlapping CpGs. Similarly, we selected regions to evaluate the number and fraction of annotated CpGs by genomic feature (i.e., CpG islands or shores—2 kb flanking the islands) and by gene part (i.e., promoter, exon, intron), and the overlap, coverage, and concordance of genomic annotations. We note that emerging epigenomic features, such as enhancers, are not considered in this analysis [32]. We used the median absolute deviation (MAD), a robust measure of variability insensitive to outliers, to estimate the statistical dispersion in methylation level comparisons.

### Computational analysis

Illumina’s CASAVA 1.8.2 was used to generate fastq files from basecalls. Raw fastq reads were processed by a custom pipeline that consists of: (1) filtering raw fastq reads for pass filter reads, (2) trimming adapter sequence by FLEXBAR [33], (3) genomic alignments performed using Bismark [34] and Bowtie [35] to reference human genome hg19, and (4) methylation calling by a custom PERL script [25]. Custom analysis scripts were written in R (version 3.3.0 [36]), including packages: Bioconductor—Biobase version 2.32.0 [37], GenomicRanges package version 1.24.0 [38], Beeswarm package version 0.2.3 [39], Affy package version 1.50.0 [40], BSgenome.Hsapiens.UCSC.hg19 package version 1.4.0 [41], PreprocessCore package version 1.34.0 [42], and the UpSetR package version 1.2.0 [43] were used to perform the analysis. Genomic features were acquired as in [24] and processed by methylKit [44] to compute each feature’s coordinates. Genomic annotations (refseq gene models and CpG islands) were downloaded from the UCSC genome browser [45]: exon, intron, and intergenic regions were defined as per gene models, promoters were defined as 2 kb upstream from the transcription start site (TSS), and CpG shores were defined as 2 kb flanking the CpG islands.

## Results

### Comparison of predicted coverage

**Fig. 1:**
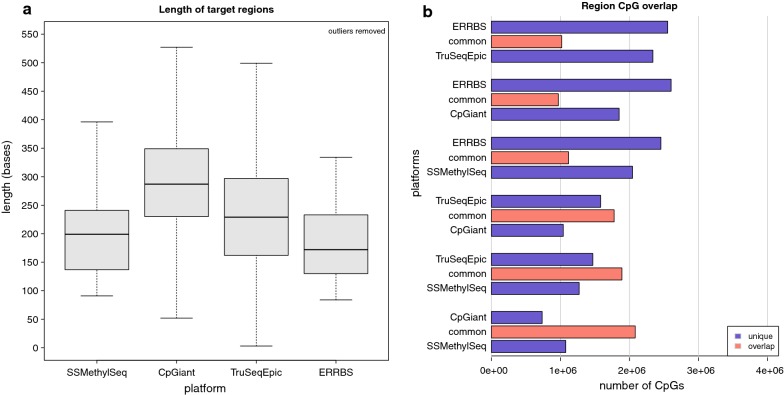
Length and overlap of design regions and MspI predicted regions. **a** Boxplot showing the distributions of targeted region’s lengths. Region lengths and CpG sites, for the capture methods, were extracted from the designed regions provided by the manufacturer and for ERRBS, extracted from fragments 84–334 bp where the genome was computationally digested with MspI. SSMethylSeq and ERRBS show similar region proportions, while CpGiant regions are generally longer and both CpGiant and TruSeqEpic are more variable in length. **b** Barplots showing the pairwise overlap comparison of each of the platform’s CpG coverage, number of region CpGs overlapping (red), number of CpGs unique to platform regions (blue). The capture platforms have a higher degree of common regions with each other than either one with ERRBS

Targeted capture techniques (SSMethylSeq, CpGiant, and TruSeqEpic) have a designed set of genomic regions, and therefore, a predicted set of CpGs covered. SSMethylSeq and TruSeqEpic are specifically designed to capture CpGs from a single DNA strand; however, some regions that contain SNP are double stranded in the TruSeqEpic platform. ERRBS, CpGiant, and WGBS-PBAT capture CpGs from both DNA strands. The ERRBS protocol is considered targeted to the extent that the restriction digest produces consistent genomic fragments (although non-MspI fragments may arise due to degradation of starting material), sizes from 84 to 334 bp are isolated during the library preparation gel extraction step. Note that since the DNA for WGBS-PBAT is randomly sheared and coverage depends upon sequencing depth, there are no predicted regions and consequently WGBS-PBAT is excluded from the predicted regions coverage analysis. Region lengths and CpG sites for the capture methods were extracted from the designed regions provided by the manufacturer. For ERRBS, region lengths and CpG sites were extracted from fragments 84–334 bp where the genome was computationally digested with MspI. Overall, the distribution of region lengths among the platforms is similar. CpGiant has the least number of regions and generally longer region lengths with a similar region length distribution to TrueSeqEpic, while ERRBS has the most regions but due to the size selection, generally shorter region lengths and a similar region length distribution to SSMethylSeq (Fig. 1a and Additional file 1: Table S2). The capture platforms cover fewer CpGs common with the restriction enzyme platform (ERRBS) and more CpGs common to each other (Fig. 1b). SSMethylSeq and CpGiant share the most CpG sites, and TruSeqEpic has the most unique sites when compared to the other capture platforms. The different capture platforms cover on average 62 ± 7.5% of each others regions CpGs and around 31 ± 3% of the ERRBS regions CpGs. This confirms that there is a large set of known regions covered across the platforms and regions that are not found in the canonical CpG-rich regions of MspI fragments (ERRBS).

### Sample preparation and sequencing

The general library preparation protocol for ERRBS is enzymatic digestion of input DNA with the methylation insensitive MspI enzyme followed by addition of adapters and barcodes, enrichment by gel size selection for fragments 84–334 bp, bisulfite conversion, and amplification by PCR. The capture methods are similar in that targeted capture of genomic regions is performed by hybridization to probes thereby providing a direct measure of CpG methylation at predefined regions. However, the capture methods differ in various important aspects. First, the SSMethylSeq and TruSeqEpic platforms capture and measure methylation levels from predominantly one DNA strand. Second, in CpGiant’s protocol, bisulfite conversion is performed before hybridization to oligonucleotides whereas in SSMethylSeq and TruSeqEpic, bisulfite conversion is performed following genomic capture. Hence, the CpGiant probe design must capture the various combinations of DNA sequences that can result from bisulfite conversion. In contrast to the targeted approaches WGBS-PBAT relies on bisulfite breakage of genomic DNA. Biases and artifacts from bisulfite sequencing have been well documented [46]. These mostly stem from an inherent bias in PCR amplification of GC-rich containing fragments over AT-rich ones and from the amount of DNA degradation after bisulfite conversion. Additionally, the presence of uracil (a result of the deamination of cytosine) stalls replicative DNA polymerases. These biases can be minimized by a controlled bisulfite conversion step and the choice of a uracil insensitive DNA polymerase. For this study we note that although the methods used were different, all techniques employed bisulfite conversion reagents from Zymo Research and all used uracil tolerant DNA polymerases. All libraries were made from human lung fibroblast IMR90 cell line DNA and prepared as described in the materials and methods with prominent differences outlined in Table 1.

### Sequencing and alignment characteristics

Generally, all platforms produced similar sequencing results with no noticeable bias or reduced quality scores. The number of clusters and number of pass filter reads produced a typically consistent number of usable reads for all samples, see Additional file 1: Table S3 for more details. It should be noted that Illumina’s TruSeqEpic is the latest methylation capture platform to be launched, and as such, has been thoroughly optimized with regards to the sequencing depth required for maximum CpG coverage of the regions represented. Therefore, TruSeqEpic libraries were sequenced per manufacturer’s instructions aiming for ~ 40× coverage.

**Fig. 2:**
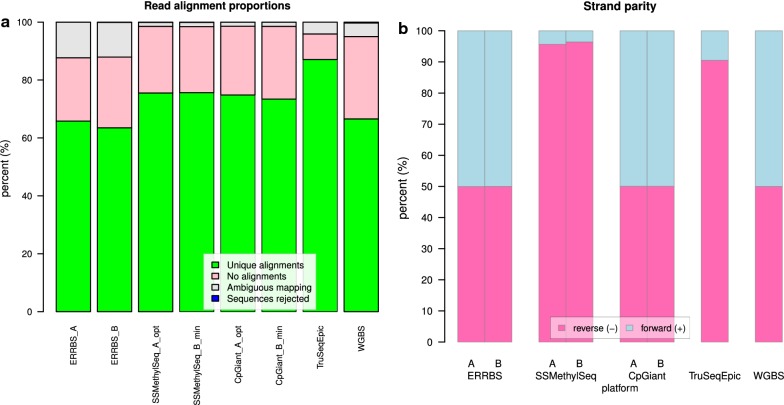
Read alignment and strand parity. **a** Percent alignment of uniquely aligned reads (green), ambiguously mapped reads (gray), reads with no alignment (pink) and rejected reads (blue). **b** The fraction of CpG’s covered at ≥ 10× coverage grouped by strand, forward/+ strand (blue) and the reverse/− strand (pink). The strand specific protocols of SSMethylSeq and TruSeqEpic platforms are evident by the high proportion of reads mapped to the reverse strand. Note that for TruSeqEpic analysis we combined the sequencing results of two libraries and for WGBS-PBAT we combined the results of two sequencing lanes to obtain a higher sequencing coverage

Consistent with other bisulfite converted samples the number of uniquely mapped reads was ~ 72.8%, SD = 7.5% (Fig. 2a). Across all platforms, a mean of 22.3%, SD = 5.8% of reads were not aligned and ambiguously mapped reads showed a larger proportion for ERRBS (~ 12.1%), than WGBS-PBAT (4.6%), SSMethylSeq (1.4%), CpGiant (1.4%), or TruSeqEpic (4.1%) (Fig. 2a). These observations indicate the propensity of designed platforms to avoid repetitive regions or paralogous regions in contrast to ERRBS where no such selection is possible.

### Strand methylation symmetry

**Fig. 3:**
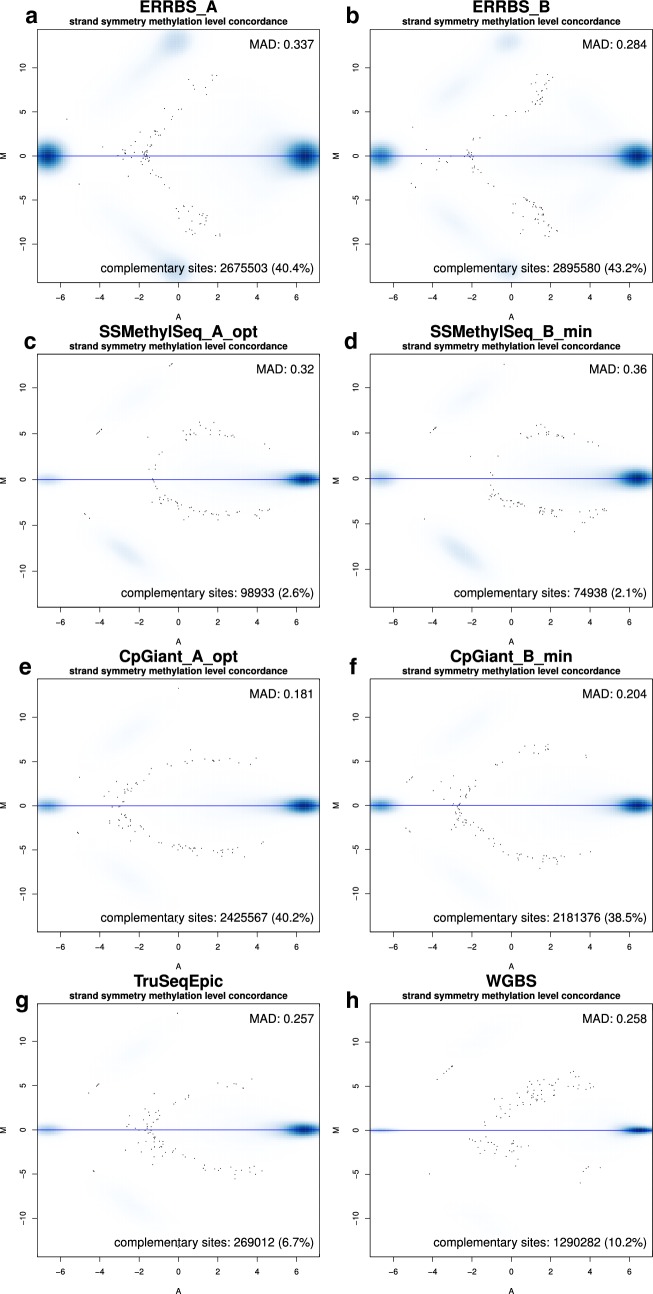
Strand symmetry of methylation values MA-plots. MA-plots of the log average of the methylation levels (*A*) on the *x*-axis and log ratio of the methylation levels (*M*) on the *y*-axis, between complementary CpG positions. Median absolute deviation (MAD) values are used to evaluate the agreement in methylation levels. The bimodal nature of methylation patterns (mostly unmethylated or methylated) is reflected in the high density at both ends of the *x*-axis. The artificially discordant sites introduced during ERRBS library preparation are identified as increased density off the center line at the low methylation values (at *A* < 0 range). **a** ERRBS_A, **b** ERRBS_B, **c** SSMethylSeq_A, **d** SSMethylSeq_B, **e** CpGiant_A, **f** CpGiant_B, **g** TruseqEpic, and **h** WGBS-PBAT

Maintenance of symmetric methylation patterns across complementary CpG sites is required to preserve methylation patterns across cellular divisions. In principle, measuring methylation levels on one strand is sufficient to infer the cellular methylation state. Indeed, the SSMethylSeq and TruSeqEpic platforms are designed to capture CpG sites from predominantly one strand, and CpGiant, ERRBS, and WGBS-PBAT cover CpG sites from both strands (Fig. 2b). We note that this is different from measuring different methylation between two parental alleles, which requires genomic phasing information and is not considered in this analysis. To validate that methylation levels are strand symmetric, we compared the methylation values between complementary CpG sites with minimal 10× coverage on both strands (Fig. 3). About 40% of the CpG sites covered on both strands were complementary for ERRBS and CpGiant, and fewer sites covered being complementary for WGBS-PBAT (~ 10%) (Fig. 3a, b, e, f, h). For completeness, the concordance of complementary CpG sites for TruSeqEpic (~ 7%) and SSMethylSeq (2–3%) are shown in Fig. 3c, d, g. We confirmed strong agreement in methylation values for those complementary CpG sites, in all samples and all platforms supporting the notion of symmetric methylation (Fig. 3, mean MAD = 0.28, SD = 0.06). We observed a discordance in ERRBS where a small set of hemi-methylated CpG sites (Fig. 3a, b), displayed inconsistent methylation values (*Δ* > 99, 3.8% of sites ERRBS_A, 1.0% in ERRBS_B at 10× coverage). This methylation discordance is a consequence of the ERRBS library preparation where the MspI staggered cut sites are de novo (in vitro) filled with C′ and G′ to generate blunt ends. The 3′ fill-in introduces unmethylated C’s at those loci and the effects of this appear to be consistent as described previously by Stockwell et al. [47]. As a result, methylated CpGs at the restriction sites (i.e., at the ends of sequences fragments) are discordant. Since the fraction of discordant sites is small, we have kept them in the analysis.

Having established that the methylation levels are concordant in both DNA strands and in order to maintain equal evaluation of CpG capture and methylation levels among all platforms, since the platforms differ whether they capture information from one DNA strand or both DNA strands, all subsequent analyses are based on CpG-units. We define CpG-units as CpG sites with ≥ 10 spanning reads regardless of which strand the reads are mapped (i.e., complementary CpG sites are combined into one unit and then filtered for  ≥ 10× coverage).

### CpG-unit coverage and target region coverage

**Fig. 4:**
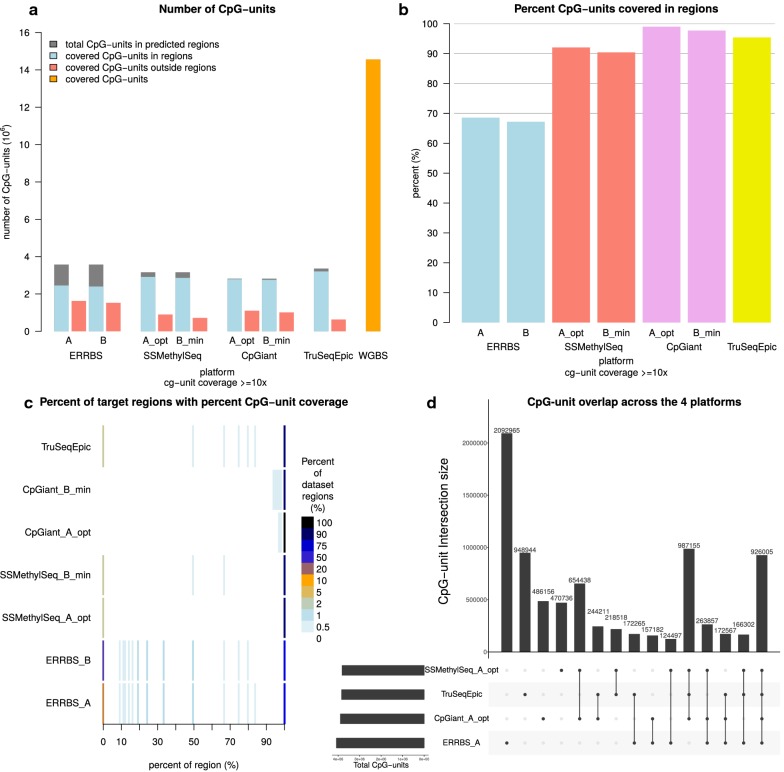
Platform CpG-unit region coverage and CpG-unit overlap. **a** Number of CpG-units identified in targeted and off-target regions by each platform. WGBS-PBAT (orange) covers ~ 14.4 M CpG-units; however, there is no notion of targeted regions in this platform. The targeted platforms predicted total CpG-units are depicted as gray bar and coverage of the CpG-units in the predicted regions (on-target) are shown in blue. CpG-units outside the predicted set (off-target) are shown in red bars. **b** Barplot of the percent recovery of targeted CpG-units. **c** Density plots showing the portion of the regions covered by the data where the *y*-axis is the length of the region from 0 to 100% and the color scale is fraction of CpG-units covering a location of the region. Primarily the end of the region is covered by all methods, however ERRBS shows abundant coverage in the start of the region and to a lesser degree throughout the region. These plots demonstrate that the reduced number of recovered CpG-units in ERRBS relative to the other platforms is attributed to increased number of missed CpG-units shown as increased density at 0% region. **d** Overlap of CpG-units across the four platforms. The UpSet visualization technique [43] for set intersections is displayed as matrix layout. Horizontal bars on the lower left indicate the total number of annotated CpG-units in the set. Dark circles in the matrix (lower right) indicate sets that are part of the intersection. Bars in the main plot area (upper right) indicate the number of intersecting CpG-units for the sets represented by the dark circles

We next evaluated the number of CpG-units covered and the mean coverage of all CpG-units that were sequenced at 10x depth or more. ERRBS, SSMethylSeq, CpGiant (each sequenced at on average 121 M paired-end reads), and TruSeqEpic (sequenced at ~ 54 M paired-end reads) platforms cover on average 3.84 M CpG-units, SD = 0.15 M at mean coverage of 105.1 reads, SD = 32.4 (Fig. 4a, b and Additional file 1: Figures S1 and S2). In contrast, WGBS-PBAT sequenced at ~ 300 M paired-end reads covered 14.4 M CpG-units (~ 50% of the 28 M total CpG-units in the genome) at a mean coverage of 19x, demonstrating that achieving comparable coverage by WGBS-PBAT is significantly more costly than targeted platforms. Therefore, these results confirm that while WGBS-PBAT profiles a large portion of CpG-units, the targeted platforms provide a cost efficient way to interrogate a limited, yet potentially most informative, set of CpG-units at considerably reduced cost. Next, we evaluated the extent of coverage in the targeted regions and the fraction of their CpGs identified. Here, we extracted the CpG-units from the regions and looked at the coverage for each sample. While region coverage and the coverage of CpG-units within the targeted regions are high, we observed that a large fraction of a sample’s CpG-units are outside the targeted regions, roughly 20–30% for the capture platforms and ~ 40% for ERRBS (Fig. 4a). We observed that ERRBS covers on average 67.9% of its predicted CpG-units in its 84–334 bp regions. CpGiant, SSMethylSeq, and TruSeqEpic cover on average 98.3, 91.2, and 95.3% of their expected CpG-units in their capture regions, respectively (Fig. 4b). In the case of ERRBS, this is expected since restriction digestion and gel isolation can be variable. In the case of capture methods, this may indicate either cross-hybridization of the probes to other genomic locations or hybridization of longer fragments. Since there are typically several CpG-units within a region, we looked at the distribution of the fraction of CpG-units covered per region and found that nearly all CpG-units in targeted regions are covered, although for ERRBS, and to a much lesser degree SSMethylSeq and TruSeqEpic, several CpGs in targeted regions are not represented presumably due to coverage bias (Fig. 4c).

### CpG-unit overlap and methylation levels concordance

**Fig. 5:**
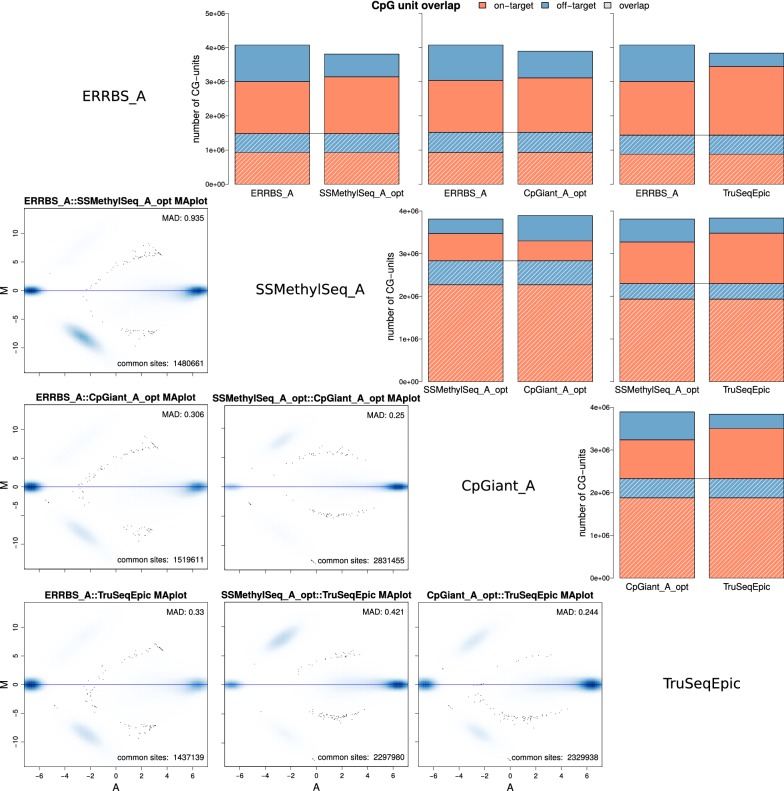
Inter-platform CpG-unit overlap and methylation levels concordance. The upper triangle shows barplots for the number of overlapping CpG-units between any two samples for the portion of off-target non-overlapping (blue), on-target non-overlapping (salmon), off-target overlapping (shaded blue) on-target overlapping (shaded salmon). The lower triangle shows MA-plots of common CpG-units between any two samples, where M is the log ratio of the methylation levels and A is the average log methylation level from the two platforms. There are blue clouds that at log scale show the variance in methylation at low levels. See Additional file 1: Figure S3 for all pairwise comparisons

We compared the overlap of CpG sites in each platforms expected regions and found there was considerable overlap of CpG loci among the capture platforms (~ 62%) and less overlap with the restriction digest platform (~ 31%). Our experimental results confirm this: the average portion of common CpG-units covered by all four platforms is ~ 29 ± 0.7% (Fig. 4d). When comparing the pairwise overlap of shared CpG-units across all samples, we observe high overlap between intra-platform (within same platform) replicates with median number of shared CpG-units ~ 3.58 M, median overlap 92.1% and high methylation level concordance with mean MAD = 0.23 and SD = 0.05 (Additional file 1: Figure S2 and Table S4). Among cross-platform comparisons, we observed reduced overlap as expected by their different designed targets with a mix of on-target and off-target sites overlapping; median number of shared CpG-units ~ 1.5 M, median overlap 39.4% (Fig. 5 upper triangle, Additional file 1: Figure S2 and Table S4). Methylation levels of common CpG-units across all platforms are highly concordant indicating that methylation level measurements are consistent and reproducible among the different platforms with average MAD = 0.39 and SD = 0.22 (Fig. 5 lower triangle, Additional file 1: Figure S2 and Table S4). Inter-platform (across all platforms) concordance was slightly lower than intra-platform concordance with mean MAD = 0.42 and SD = 0.23 (Fig. 5 lower triangle, Additional file 1: Figure S2 and Table S4). These results demonstrate that while the platforms differ in their capture approaches and bisulfite conversion kits (Table 1), these differences are not biasing methylation measurements. The differences among the platforms, therefore, are largely restricted to variations in targeted regions and not in methylation measurements.

### Coverage of genomic feature regions by CpG-units

**Fig. 6:**
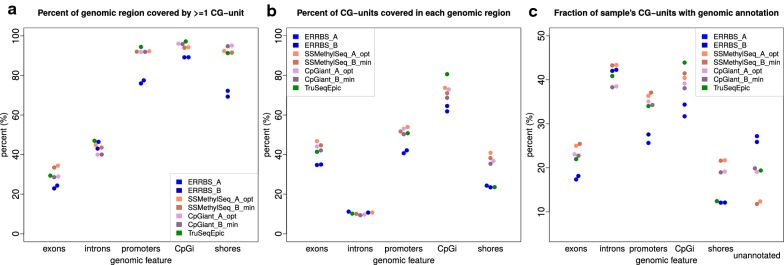
Summary of coverage and representation of annotated genomic regions by each platform. A region here is defined as a specific genomic feature. **a** The fraction of the regions covered by at least 1 CpG-unit for each sample for each region. **b** The fraction of a region’s complement of CpG-units covered for each sample for each region. **c** The fraction of a sample’s CpG-units annotated with a genomic feature

Next we were interested in what genomic features are covered by each platform and the degree of coverage. Analogous to the previous analysis, here we define a region as being one of:  exon, intron, promoter, CpG island, or CpG shore, and coverage is a genomic region that contains at least one detected CpG-unit. It should be noted that the genomic feature annotations are not mutually exclusive and that some CpG-units are annotated by more than one category. Naturally, the targeted platforms focus on genomic regions known to play important roles in epigenetic regulation such as promoter regions, CpG islands, and CpG shores, while ERRBS covers the same regions, but to a lesser degree (Fig. 6a). Moreover, each platform covers similar proportions of CpG-units in the annotated genomic regions and we observe TruSeqEpic covering slightly more CpG islands and fewer CpG shores than the other capture platforms (Fig. 6b). Conversely, we looked at the number of CpG-units that are annotated and observed similar trends for the four platforms with ERRBS having the largest proportion of unannotated CpG-units (~ 27%, Fig. 6c). Overall, no platform appears significantly enriched for specific genomic regions with the qualification that TruSeqEpic is slightly less represented in CpG shores and ERRBS is slightly less represented in promoters, CpG islands and CpG shores, while having more unannotated CpG-units.

### Overlap of CpG-units annotated with a genomic feature

**Fig. 7:**
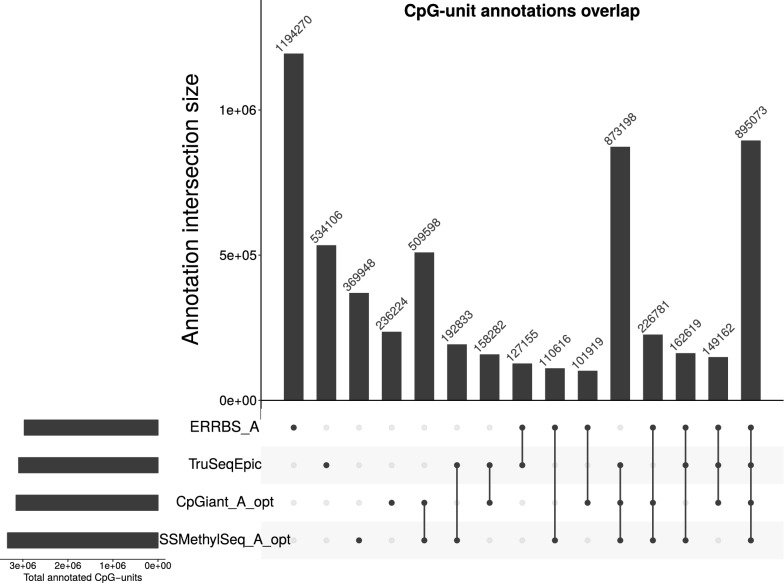
CpG-unit annotations overlap. Overlaps of all CpG-unit annotations across all platforms. The UpSet visualization technique [43] for set intersections is displayed as matrix layout. Horizontal bars on the lower left indicate the total number of annotated CpG-units in the set. Dark circles in the matrix (lower right) indicate sets that are part of the intersection. Bars in the main plot area (upper right) indicate the number of intersecting CpG-units for the sets represented by the dark circles. Roughly 29% of the annotations are common to all four suggesting that while there may be similar proportions of CpG-unit annotations (i.e., they may be covering similar regions), they are covering different loci within those regions

We evaluated the overlap of annotated CpG-units to determine whether any platform is enriched for a specific genomic feature (e.g., exons, introns, promoters, CpG islands and shores), where a CpG-unit is counted as annotated if it has one or more annotations. Comparing the overlap in annotations across platforms, we see a similar grouping as above with the methylation levels, intra-platform overlap is high (mean overlap 93.7%, SD = 2.9%), and inter-platform overlap is lower (mean 56.3%, SD = 14%) (Fig. 7 and Additional file 1: Figures S3–S8). Thus, we observed similar proportions of genomic region coverage across all platforms, but lower overlap of annotated CpG-units suggesting coverage of different loci within those regions.

## Discussion

We performed a systematic analysis of the characteristics of four prominent platforms for measuring DNA methylation levels: ERRBS, Agilent SureSelect Methyl-Seq (SSMethylSeq), Roche NimbleGen SeqCap Epi CpGiant (CpGiant), and Illumina TruSeq-Methyl capture EPIC (TruSeqEpic) with the goal of identifying whether one method outperforms the others in any characteristic. We assessed the expected region coverage for SSMethylSeq, CpGiant, TruSeqEpic and ERRBS and observed that, overall, each platform covers a large fraction of its targeted regions, and the CpG sites covered by each platform are largely distinct from each other. SSMethylSeq and CpGiant cover roughly 70% of the same CpGs, TruSeqEpic shares roughly 40–50% of the same CpGs as the other capture methods, while ERRBS has only ~ 30% overlap with any other capture method. SSMethylSeq, CpGiant, and TruSeqEpic cover greater than 90% of CpGs in their designed capture regions and ERRBS covers roughly 70% of CpGs in its expected fragments. Furthermore, methylation levels of common CpG-units across all platforms are highly concordant.

Finally, each platform covered roughly the same proportions of genomic features (CpG islands, shores, promoters, exons, and introns), but profiled different CpG sites within those regions. In addition to the differences in the targeted CpG positions, there are also differences in the library preparation protocols. ERRBS can be performed using small amounts of starting material (as little as 75 ng), whereas SSMethylSeq requires 1 µg, TruSeqEpic 0.5 µg and CpGiant 0.25 µg of starting DNA (although those are expected to reduce with further optimization by the vendors or researchers). For example, Miura and Ito [48] have developed a modified SSMethylSeq using nanograms of starting material by amplifying bisulfite converted DNA using a PBAT approach. ERRBS and other digestion-based methods can cover certain genomic regions that are not amenable to captured by oligo designs. For example, another enzymatic approach, cuRRBS [49], allows the user to optimally design combinations of enzymes that cover regions of interest. While this method can achieve more specific targeting than ERRBS, which uses a single restriction enzyme, it is still a target enrichment protocol as opposed to targeted platforms that are based on designed probes for regions of interest, and it is limited by the number of enzymatic reactions. ERRBS provides comparable data to the capture platforms, although there is more variability among the profiled CpGs. Capture platforms are more precise and can be customized for profiling specific genomic regions of interest. For example, custom-capture platforms for studying the maize methylome and mouse neuronal methylome have been published [50–52]. In addition, we found that the SSMethylSeq capture platform can be used for methylation profiling of low-quality, degraded DNA although efficiency of capture is somewhat dependent on shearing (data not shown). This is an ideal platform for clinical samples stored as formalin-fixed paraffin-embedded (FFPE) tissue.

## Conclusions

Epigenetic state is a fundamental element of cellular development and regulation. Therefore, accurate, reproducible and cost effective approaches for profiling DNA methylation are important for advancements in biomedical research. While the cost of sequencing continues to decrease, reaching sufficient coverage for reliable measurement of CpG methylation by WGBS-PBAT is still prohibitive. We conclude from our comparative study that capture-based approaches provide comparable results and cover more precisely their intended targets than ERRBS, which is a restriction enzyme-based approach. While Agilent and Roche and Illumina all provide a commercial design, Agilent and Roche also provide the added flexibility of designing custom captures for surveying regions of interest. However, while the Illumina TruSeq Epic platform is not amenable to custom design, it might be platform of choice for studies looking to expand on the investigations from Illumina bead arrays. On the other hand, digestion-based protocols are currently cheaper and may be the only option for clinical samples where the amount of input DNA is limited. In the absence of any prior knowledge about the genomic regions of interest for a study, the choice of methylation profiling platform would be guided by cost, the amount, and quality of input DNA.

## Additional file

**Additional file 1.** **Table S1.** Library input details. **Table S2.** Target region properties and CpGs covered. **Table S3.** Sequencing details. **Figure S1.** Number of CpG-units covered, Mean and median coverage per CpG-unit. **Figure S2.** Intra- and Inter-platform CpG-unit overlap and methylation levels concordance. **Table S4.** Intra- and Inter-platform details. **Figure S3.** Overlap of exon annotation of CpG-units as UpSet plot. **Figure S4.** Overlap of intron annotation of CpG-units as UpSet plot. **Figure S5.** Overlap of promoters annotation of CpG-units as UpSet plot. **Figure S6.** Overlap of CpG island annotation of CpG-units as UpSet plot. **Figure S7.** Overlap CpG shores annotation of CpG-units as UpSet plot. **Figure S8.** Overlap of unannotated CpG-units as UpSet plot.

## Abbreviations

5mC: 5-methylcytosine
BS-seq: bisulfite sequencing
CpGiant: Roche NimbleGen SeqCap Epi CpGiant
CpGi: CpG island
ERRBS: enhanced reduced representation bisulfite sequencing
GEO: gene expression omnibus
MAD: median absolute deviation
PCR: polymerase chain reaction
SSMethylSeq: Agilent SureSelect Methyl-Seq
TruSeqEpic: Illumina TruSeq-Methyl capture EPIC
WGBS-PBAT: whole-genome bisulfite sequencing-post-bisulfite adapter tagging
ATCC: American Type Culture Collection
FFPE: formalin-fixed paraffin-embedded
