# Supplementary material for: “Same difference”: comprehensive evaluation of four DNA methylation measurement platforms
Source: Epigenetics Chromatin. 2018 May 25;11:21. doi: 10.1186/s13072-018-0190-4 (PMC5970534; doi:10.1186/s13072-018-0190-4)

Additional file 1.

Table S1. Library input details.

Sample ID	Sample Input Concentration	pM of Library loaded in sequencer	Yield (Mbases)	% of>=Q30 Bases (PF)	Mean Quality Score (PF)
ERRBS_A	75 ng	6.5	23,632	88.84	34.51
ERRBS_B	75 ng	6.5	26,947	87.47	34.28
SSMethylSeqA_opt	3µg	11*	23,299	91.57	35.69
SSMethylSeqB_min	1µg	11*	19,532	93.56	36.33
CpGiant_A_opt	1µg	12**	29,385	84.16	33.58
CpGiant_B_min	0.25µg	12**	23,304	85.24	33.89
TruSeqEpic[1]	0.5µg	10*	5,719[2]	95.39	38.17
TruSeqEpic[1]	0.5µg	10*	5,265[2]	95.46	38.19
WGBS[3]	100ng	7	30,457	88.06	34.68
WGBS[3]	100ng	7	28,623	89.11	34.98
		average	21,616	89.89	35.43
		median	23,468	88.98	34.83
		stdev	9,130	3.99	1.66

*underclustered, **overclustered, [1, 2]Yield represents ~33M reads since libraries were sequenced as a 4-plex as per Illumina's recommendation - for analysis purposes individual libraries were combined to achieve ~40x coverage, [3]Same library was sequenced over 2 lanes to achieve ~20x coverage.

Table S2. Target region properties and CpGs covered. Capture methods (SSMethylSeq, CpGiant and TruSeqEpic) have a predicted set of regions and CpGs covered. In practice, ERRBS can be considered a targeted method since restriction digestion produces consistent genomic fragments and, during library preparation, certain sized fragments (84-334bp) are isolated.

Platform	Capture region (Mbases)	Number of regions	Number of CpGs (10^6) (single strand)	Average target CpGs covered (%)
CpGiant	80.5	240,131	2.8	98.5 +/- 0.92
SSMethylSeq	84.3	347,913	3.7	91.5 +/- 1.18
TruSeqEpic	107.8	437,792	3.3	95.4
ERRBS (MspI 84-334bp)	100.6	548,203	3.6	67.9 +/- 0.97

Table S3. Sequencing details. Data represents one sequencing lane on a HiSeq2500 in a High Output mode (version 3 chemistry), except for TruSeqEpic which was sequenced on a HiSeq2500 Rapid Mode.

Sample	% of raw clusters per lane	# usable cluster	# Reads	% PF	# usable reads
ERRBS A	85.02	20,092	309,997,646	75.48	233,986,223
ERRBS B	87.54	23,589	331,125,710	80.57	266,787,985
SSMethylSeqA_opt*	84.17	19,611	250,935,060	91.93	230,684,601
SSMethylSeqB_min*	97.09	18,964	202,372,642	95.56	193,387,297
CpGiant_A_opt**	83.94	24,666	443,905,444	65.54	290,935,628
CpGiant_B_min**	58.91	13,728	316,245,466	72.96	230,732,692
TruSeqEpic[1*]	21.06	1,204	29,276,795	96.71	28,313,588
TruSeqEpic[1*]	19.39	1,021	26,955,672	96.69	26,063,439
WGBS A[2]	100	30,457	370,610,744	81.37	301,565,962
WGBS A[2]	100	28,623	329,670,820	85.96	283,385,037
average	73.7	18,196	261,109,600	84.3	208,584,245
median	84.6	19,851	313,121,556	83.7	232,359,458
stdev	30.6	10,219	138,470,127	10.9	101,147,156

[*]underclustered, [**]overclustered, [1]Note, The TruSeqEpic lane was sequenced according to the manufacturers instructions which resulted to ~20x coverage. For analysis, the two individual libraries were combined to achieve ~40x coverage to make it consistent with the other platforms, [2]Same library was sequenced over 2 lanes to achieve ~20x coverage.

Figure S1. **A**) Mean CpG-unit coverage, **B**) Median CpG-unit coverage, **C**) Number of CpG-units covered, **D**) Distribution of coverage per CpG-unit.

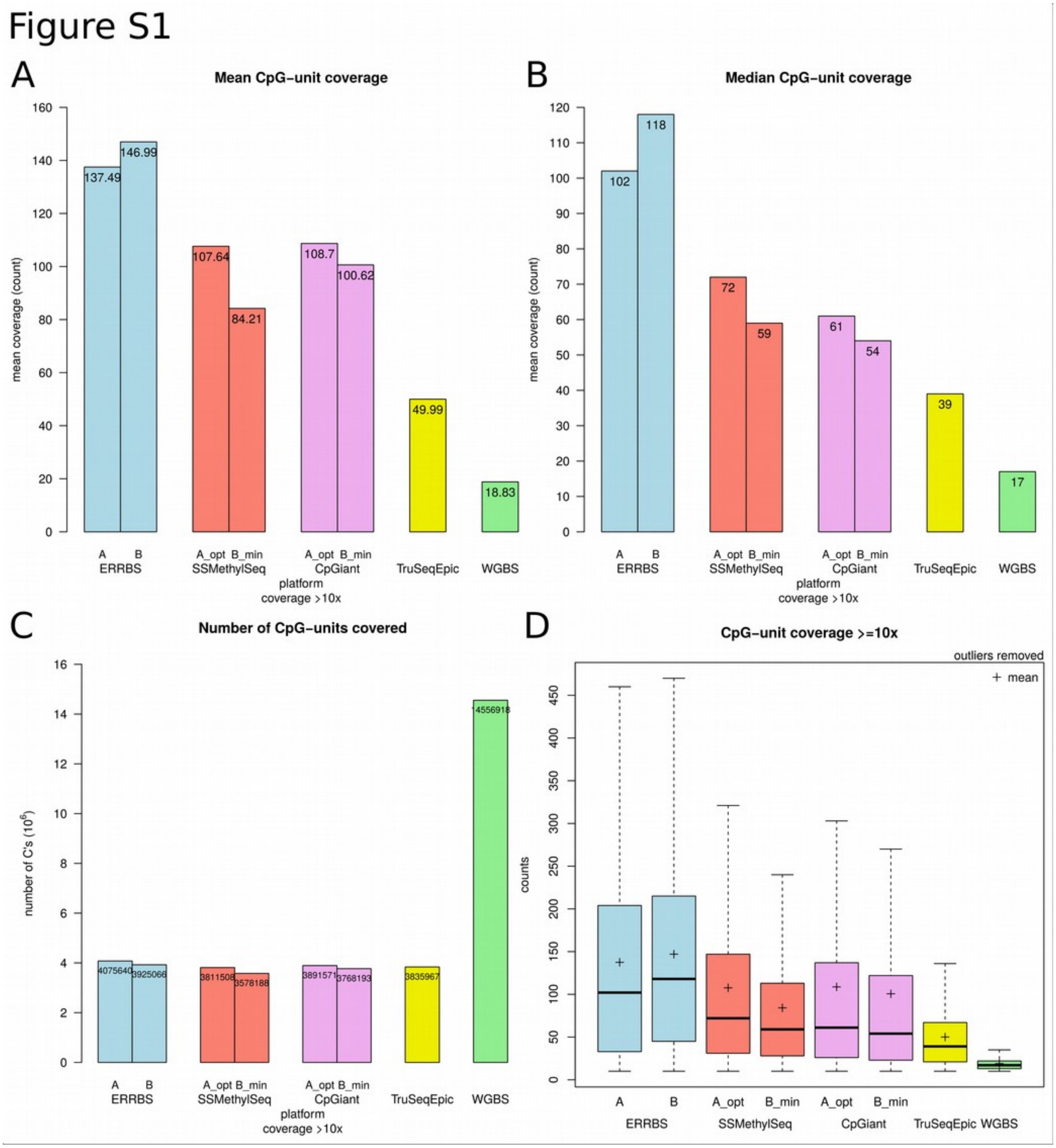

Figure S2. Intra- and Inter- platform CpG-unit overlap and methylation levels concordance.

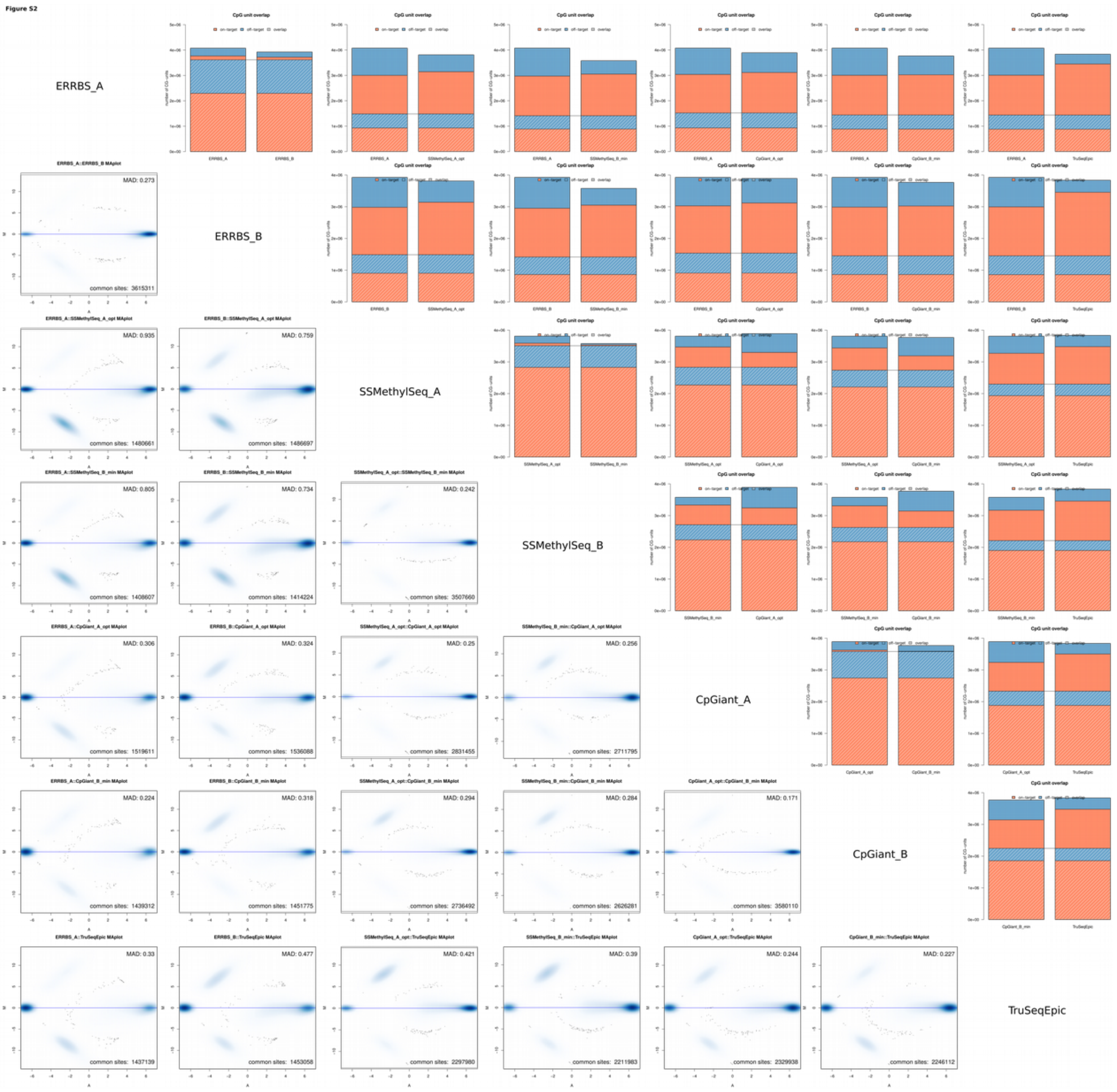

Table S4. Intra- and Inter- platform details. Columns of table: Total number of CpG-units for datasets A and B, number of shared CpG-units between datasets A and B (common), percent of sample dataset for datasets A and B, MAD value, Pearson correlation, Spearman correlation, interquartile range (IQR), and root-mean-square deviation (RMSD).

SampleA::SampleB	Total A	Total B	Common	Percent A	Percent B	Pearson	Spearman	MAD	IQR	RMSD
ERRBS_A::ERRBS_B	4,075,640	3,925,066	3,615,311	88.71	92.11	0.942	0.928	0.273	0.450	13.3
ERRBS_A::SSMethylSeq_A_opt	4,075,640	3,811,508	1,480,661	36.33	38.85	0.895	0.819	0.935	6.560	17.5
ERRBS_A::SSMethylSeq_B_min	4,075,640	3,578,188	1,408,607	34.56	39.37	0.893	0.814	0.805	5.510	17.5
ERRBS_A::CpGiant_A_opt	4,075,640	3,891,571	1,519,611	37.29	39.05	0.897	0.843	0.306	0.951	17.3
ERRBS_A::CpGiant_B_min	4,075,640	3,768,193	1,439,312	35.31	38.20	0.888	0.843	0.224	0.539	18.2
ERRBS_A::TruSeqEpic	4,075,640	3,835,967	1,437,139	35.26	37.46	0.888	0.795	0.330	0.988	16.8
ERRBS_A::WGBS	4,075,640	14,556,918	2,868,834	70.39	19.71	0.810	0.792	0.402	0.848	26.7
ERRBS_B::SSMethylSeq_A_opt	3,925,066	3,811,508	1,486,697	37.88	39.01	0.938	0.854	0.759	1.510	13.5
ERRBS_B::SSMethylSeq_B_min	3,925,066	3,578,188	1,414,224	36.03	39.52	0.936	0.847	0.734	1.300	13.6
ERRBS_B::CpGiant_A_opt	3,925,066	3,891,571	1,536,088	39.14	39.47	0.938	0.880	0.324	0.525	13.4
ERRBS_B::CpGiant_B_min	3,925,066	3,768,193	1,451,775	36.99	38.53	0.930	0.876	0.318	0.429	14.4
ERRBS_B::TruSeqEpic	3,925,066	3,835,967	1,453,058	37.02	37.88	0.932	0.823	0.477	0.685	13.1
ERRBS_B::WGBS	3,925,066	14,556,918	2,757,412	70.25	18.94	0.874	0.853	0.399	0.638	21.9
SSMethylSeq_A_opt::SSMethylSeq_B_min	3,811,508	3,578,188	3,507,660	92.03	98.03	0.981	0.936	0.242	0.328	7.69
SSMethylSeq_A_opt::CpGiant_A_opt	3,811,508	3,891,571	2,831,455	74.29	72.76	0.983	0.934	0.250	0.476	7.22
SSMethylSeq_A_opt::CpGiant_B_min	3,811,508	3,768,193	2,736,492	71.80	72.62	0.976	0.928	0.294	0.678	8.77
SSMethylSeq_A_opt::TruSeqEpic	3,811,508	3,835,967	2,297,980	60.29	59.91	0.977	0.887	0.421	0.943	8.3
SSMethylSeq_A_opt::WGBS	3,811,508	14,556,918	2,919,964	76.61	20.06	0.961	0.912	0.364	0.660	11.3
SSMethylSeq_B_min::CpGiant_A_opt	3,578,188	3,891,571	2,711,795	75.79	69.68	0.981	0.929	0.256	0.421	7.75
SSMethylSeq_B_min::CpGiant_B_min	3,578,188	3,768,193	2,626,281	73.40	69.70	0.973	0.923	0.284	0.546	9.17
SSMethylSeq_B_min::TruSeqEpic	3,578,188	3,835,967	2,211,983	61.82	57.66	0.974	0.882	0.390	0.748	8.69
SSMethylSeq_B_min::WGBS	3,578,188	14,556,918	2,761,518	77.18	18.97	0.958	0.908	0.348	0.567	11.7
CpGiant_A_opt::CpGiant_B_min	3,891,571	3,768,193	3,580,110	92.00	95.01	0.975	0.942	0.171	0.240	8.93
CpGiant_A_opt::TruSeqEpic	3,891,571	3,835,967	2,329,938	59.87	60.74	0.975	0.902	0.244	0.334	8.48
CpGiant_A_opt::WGBS	3,891,571	14,556,918	2,892,175	74.32	19.87	0.959	0.917	0.252	0.338	11.7
CpGiant_B_min::TruSeqEpic	3,768,193	3,835,967	2,246,112	59.61	58.55	0.968	0.902	0.227	0.313	9.7
CpGiant_B_min::WGBS	3,768,193	14,556,918	2,799,116	74.28	19.23	0.950	0.911	0.236	0.313	12.8
TruSeqEpic::WGBS	3,835,967	14,556,918	2,777,296	72.40	19.08	0.950	0.905	0.283	0.375	12.7

Figure S3. Overlap of exon annotation of CpG-units as UpSet plot.

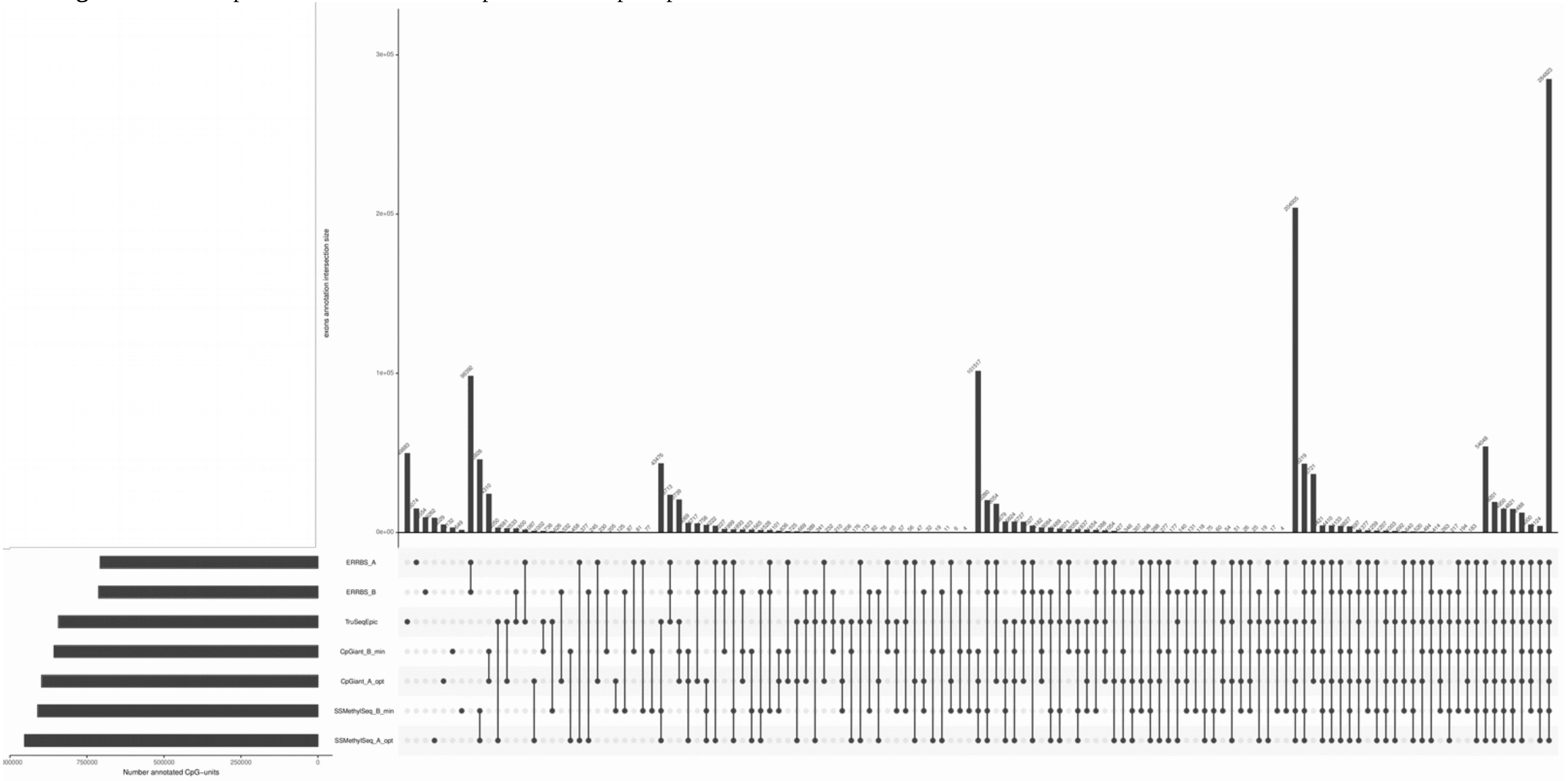

Figure S4. Overlap of intron annotation of CpG-units as UpSet plot.

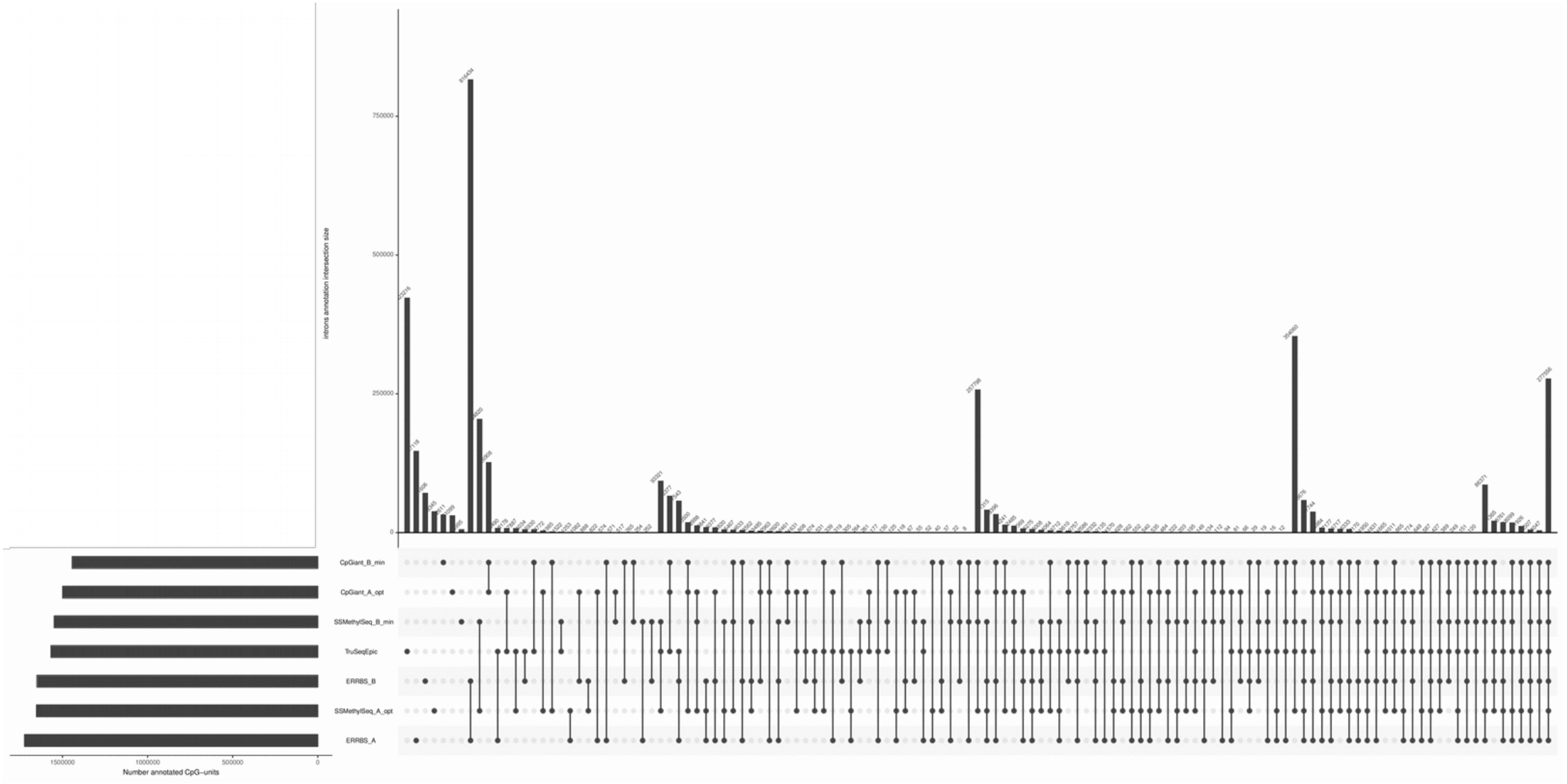

Figure S5. Overlap of promoters annotation of CpG-units as UpSet plot.

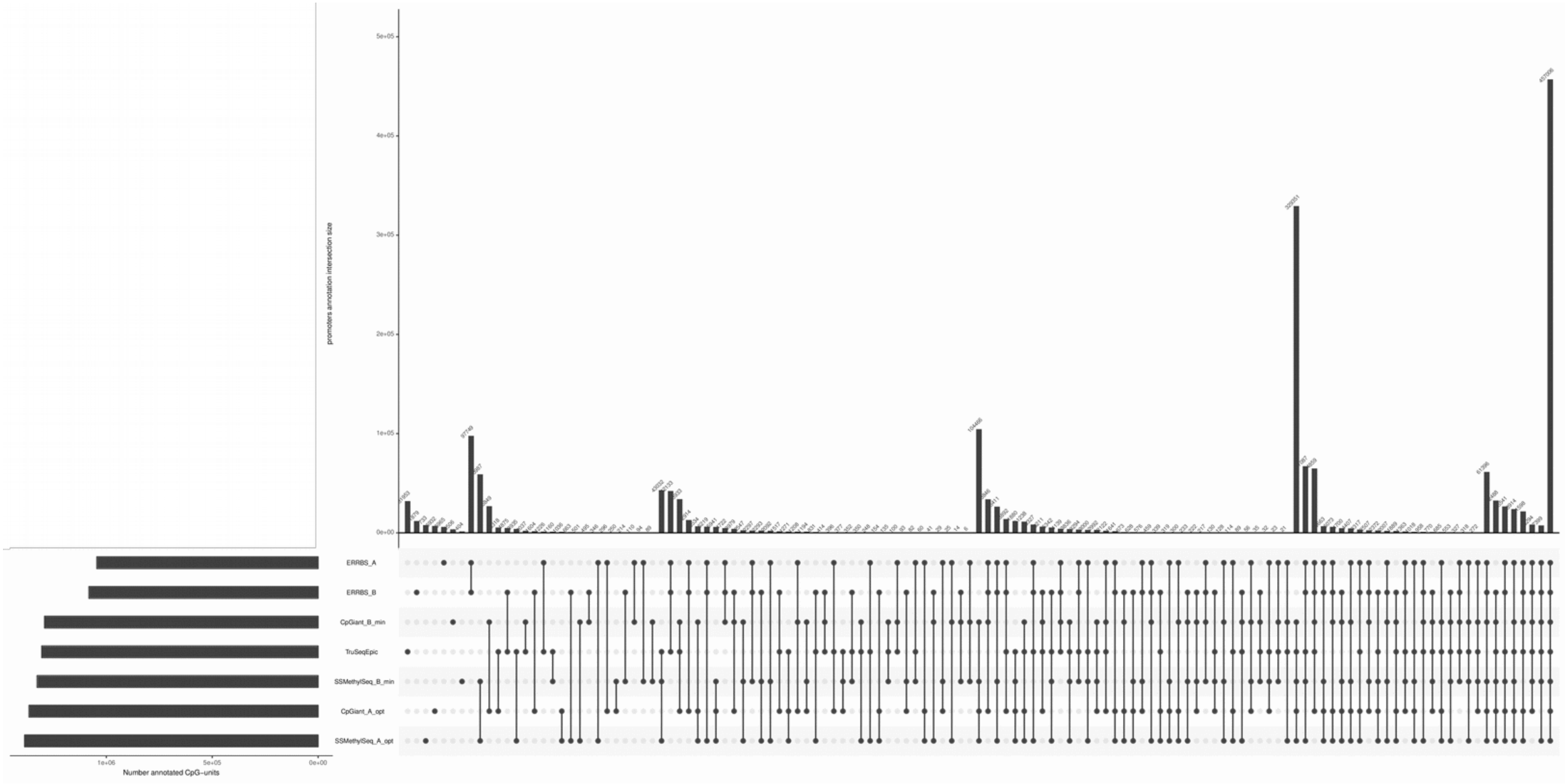

Figure S6. Overlap of CpG island annotation of CpG-units as UpSet plot.

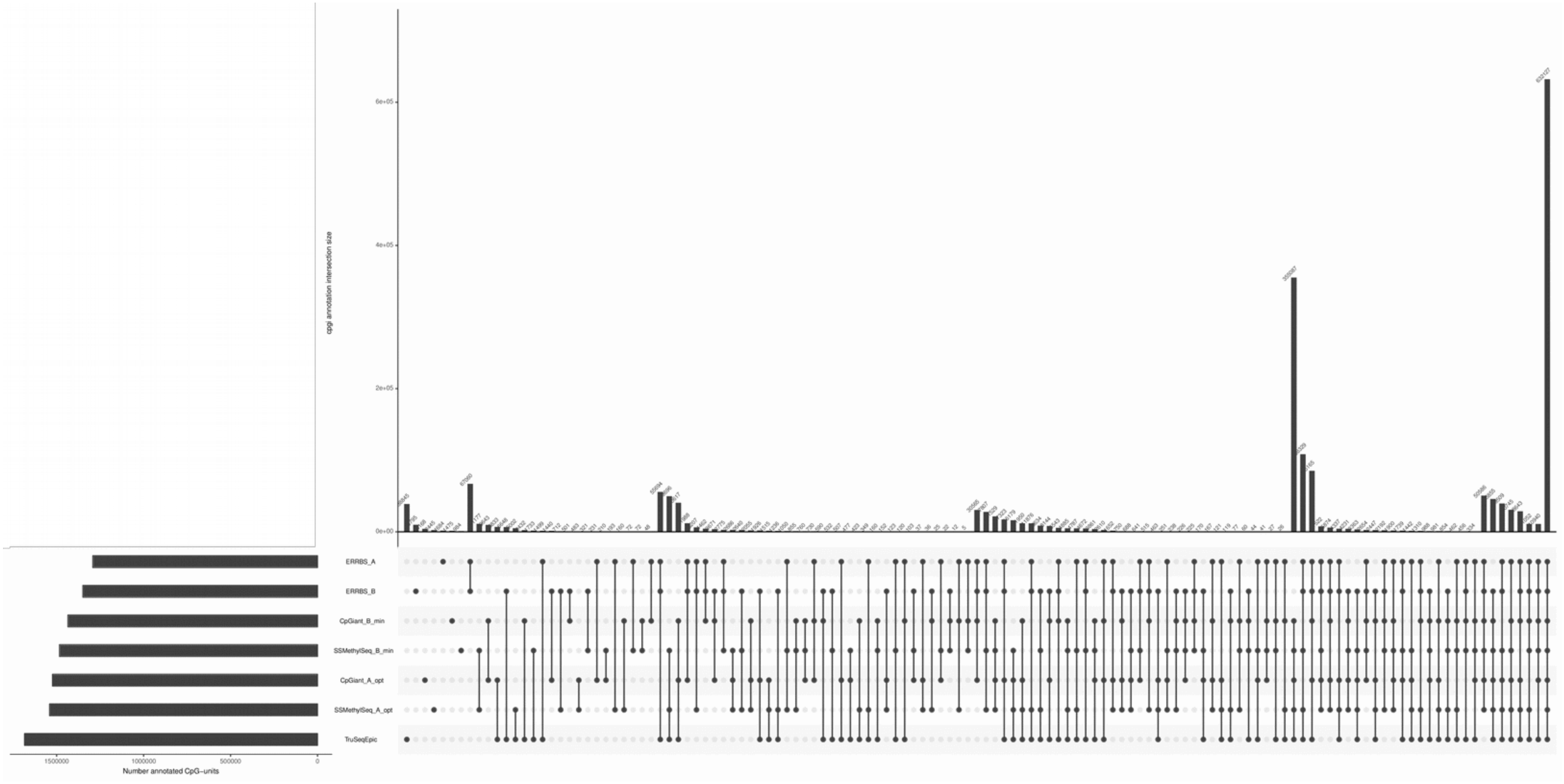

Figure S7. Overlap CpG shores annotation of CpG-units as UpSet plot.

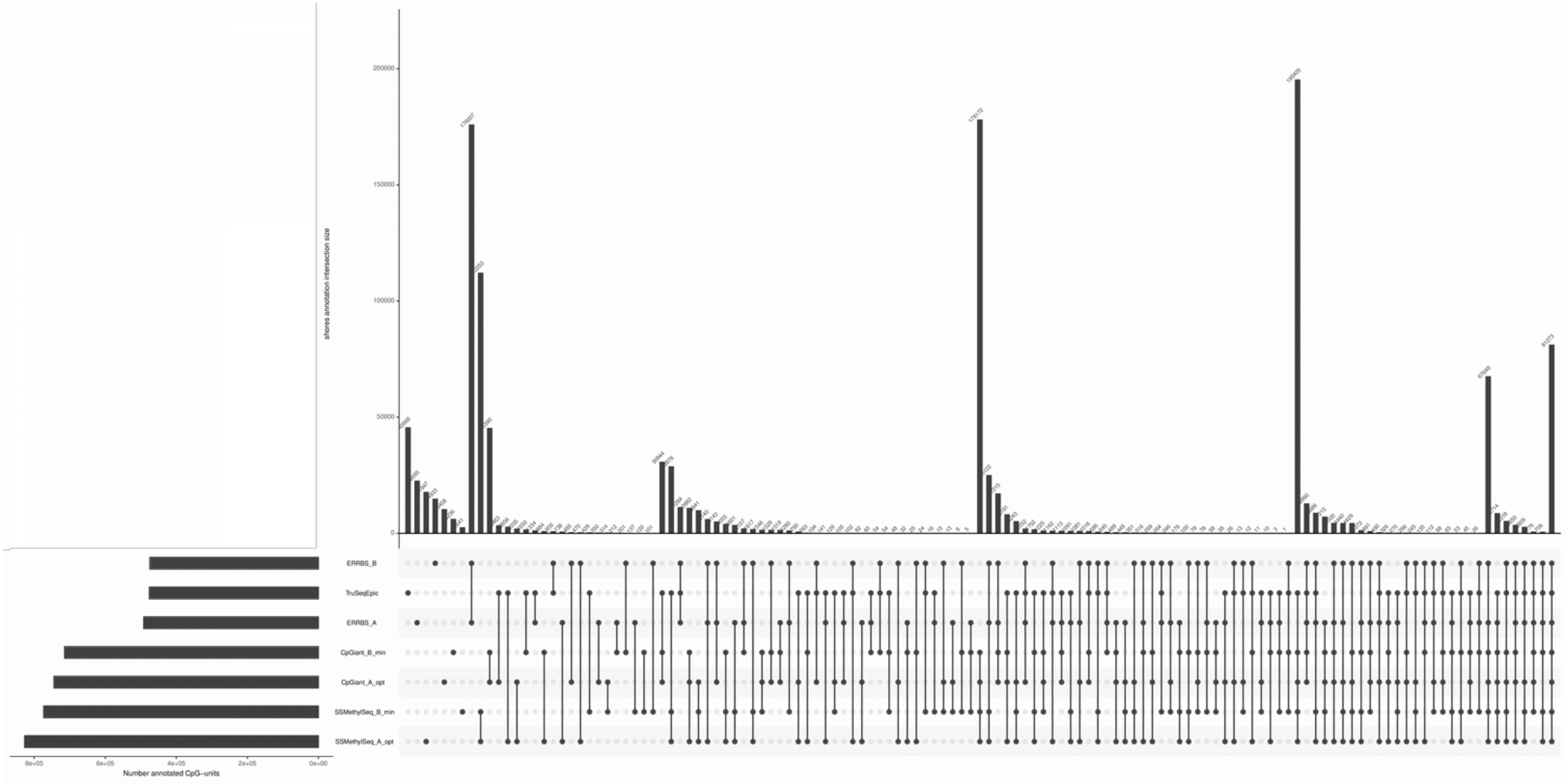

Figure S8. Overlap of unannotated CpG-units as UpSet plot.

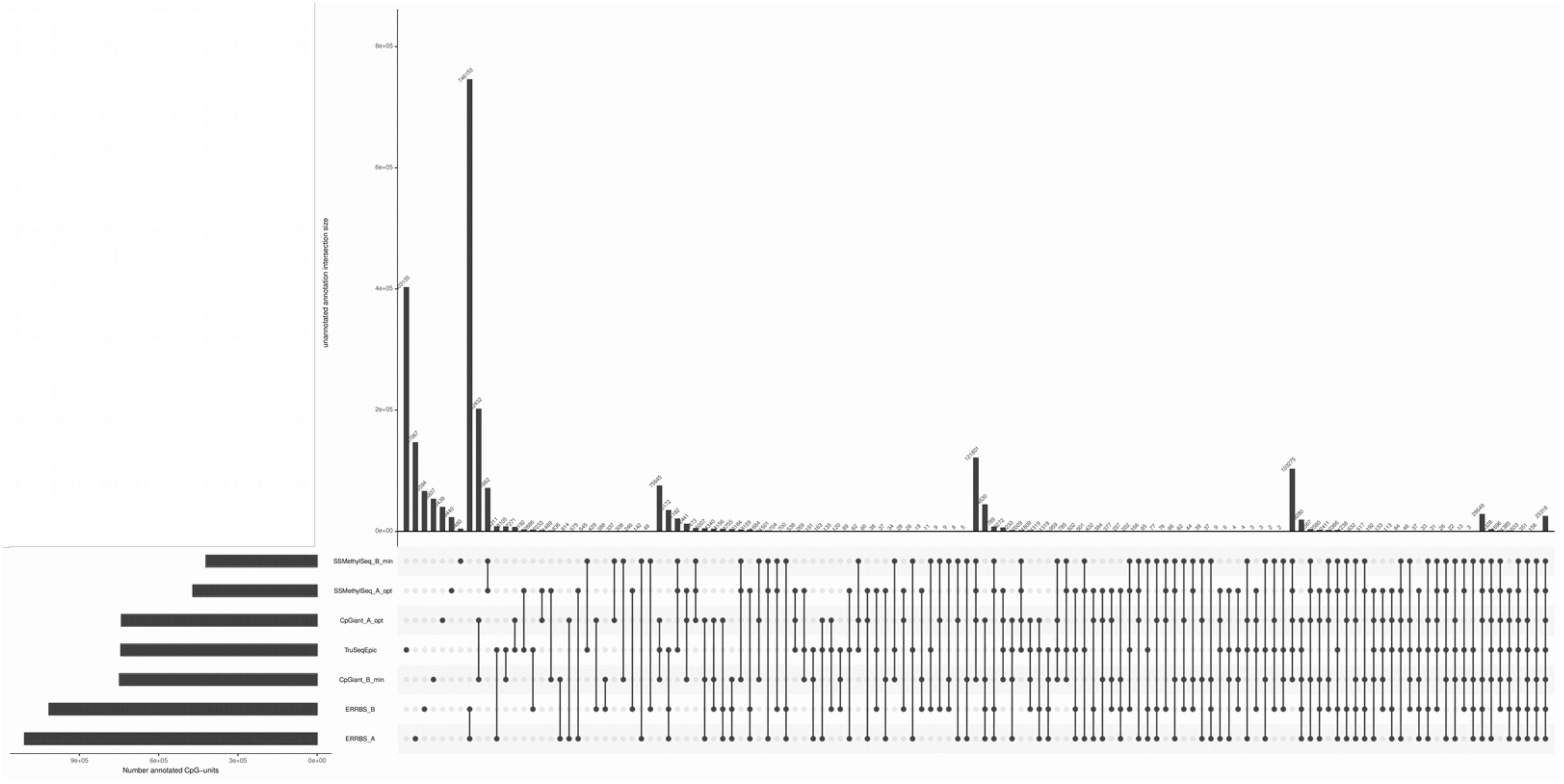

Supplement: Supplementary file 1 — Additional file 1. Table S1. Library input details. Table S2. Target region properties and CpGs covered. Table S3. Sequencing details. Figure S1. Number of CpG-units covered, Mean and median coverage per CpG-unit. Figure S2. Intra- and Inter-platform CpG-unit overlap and methylation levels concordance. Table S4. Intra- and Inter-platform details. Figure S3. Overlap of exon annotation of CpG-units as UpSet plot. Figure S4. Overlap of intron annotation of CpG-units as UpSet plot. Figure S5. Overlap of promoters annotation of CpG-units as UpSet plot. Figure S6. Overlap of CpG island annotation of CpG-units as UpSet plot. Figure S7. Overlap CpG shores annotation of CpG-units as UpSet plot. Figure S8. Overlap of unannotated CpG-units as UpSet plot. [file 13072_2018_190_MOESM1_ESM.pdf]
